# Methane Venting at Cold Heavy Oil Production with Sand (CHOPS) Facilities Is Significantly Underreported and Led by High-Emitting Wells with Low or Negative Value

**DOI:** 10.1021/acs.est.2c06255

**Published:** 2023-02-06

**Authors:** Simon A. Festa-Bianchet, David R. Tyner, Scott P. Seymour, Matthew R. Johnson

**Affiliations:** Energy & Emissions Research Laboratory, Department of Mechanical and Aerospace Engineering, Carleton University, Ottawa, Ontario Canada, K1S 5B6

**Keywords:** venting, methane, heavy oil, LiDAR, spectroscopy, unsteady, carbon pricing, emissions, marginal wells

## Abstract

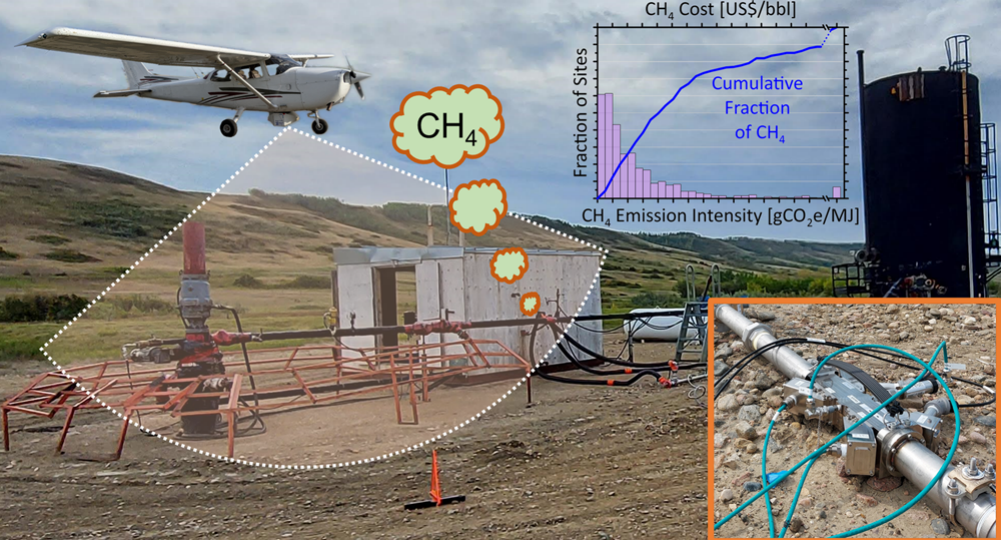

Cold Heavy Oil Production with or without Sand, CHOP(S), facilities produce a significant portion of Canada’s conventional oil. Methane venting from single-well CHOPS facilities in Saskatchewan, Canada was measured (i) using Bridger Photonics’ airborne Gas Mapping LiDAR (GML) at 962 sites and (ii) on-site using an optical mass flux meter (VentX), ultrasonic flow meter, and QOGI camera at 11 sites. The strong correlation between ground measurements and airborne GML supported subsequent detailed analysis of the aerial data and to our knowledge is the first study to directly test the ability of airplane surveys to accurately reproduce mean emission rates of unsteady sources. Actual methane venting was found to be nearly four times greater than the industry-reported levels used in emission inventories, with ∼80% of all emissions attributed to casing gas venting. Further analysis of site-total emissions revealed potential gaps in regulations, with 14% of sites appearing to exceed regulated limits while accounting for 61% of measured methane emissions. Finally, the concept of marginal wells was adapted to consider the inferred cost of methane emissions under current carbon pricing. Results suggest that almost a third of all methane is emitted from environmentally marginal wells, where the inferred methane cost negates the value of the oil produced. Overall, the present results illustrate the importance of independent monitoring, reporting, and verification (MRV) to ensure accuracy in reporting and regulatory compliance, and to ensure mitigation targets are not foiled by a collection of disproportionately high-emitting sites.

## Abstract 2

Combined aerial and ground-based measurements reveal the character and extent of methane venting from heavy oil wells. Large discrepancies with reported values are driven by environmentally marginal wells.

## 1 Introduction

Limiting methane emissions is essential to slowing global temperature rise, with the potential for critical near-term benefits in both air quality and climate forcing due to methane’s strong global warming potential and relatively short life span in the atmosphere.^1,2^ Since the COP26 meeting, more than 100 countries have signed the Global Methane Pledge,^3^ intended to rapidly curb anthropogenic methane emissions. Among the signatories, Canada has specifically committed to reducing methane emissions from the oil and gas sector by 75% from 2012 levels by 2030.^4^ The enhanced regulations required to reach this goal are in development and expected in 2023.^5^ Thus, there exists a short window of opportunity to improve our understanding of current methane emissions such that these regulations succeed in achieving their stated goal.

**Figure 1:**
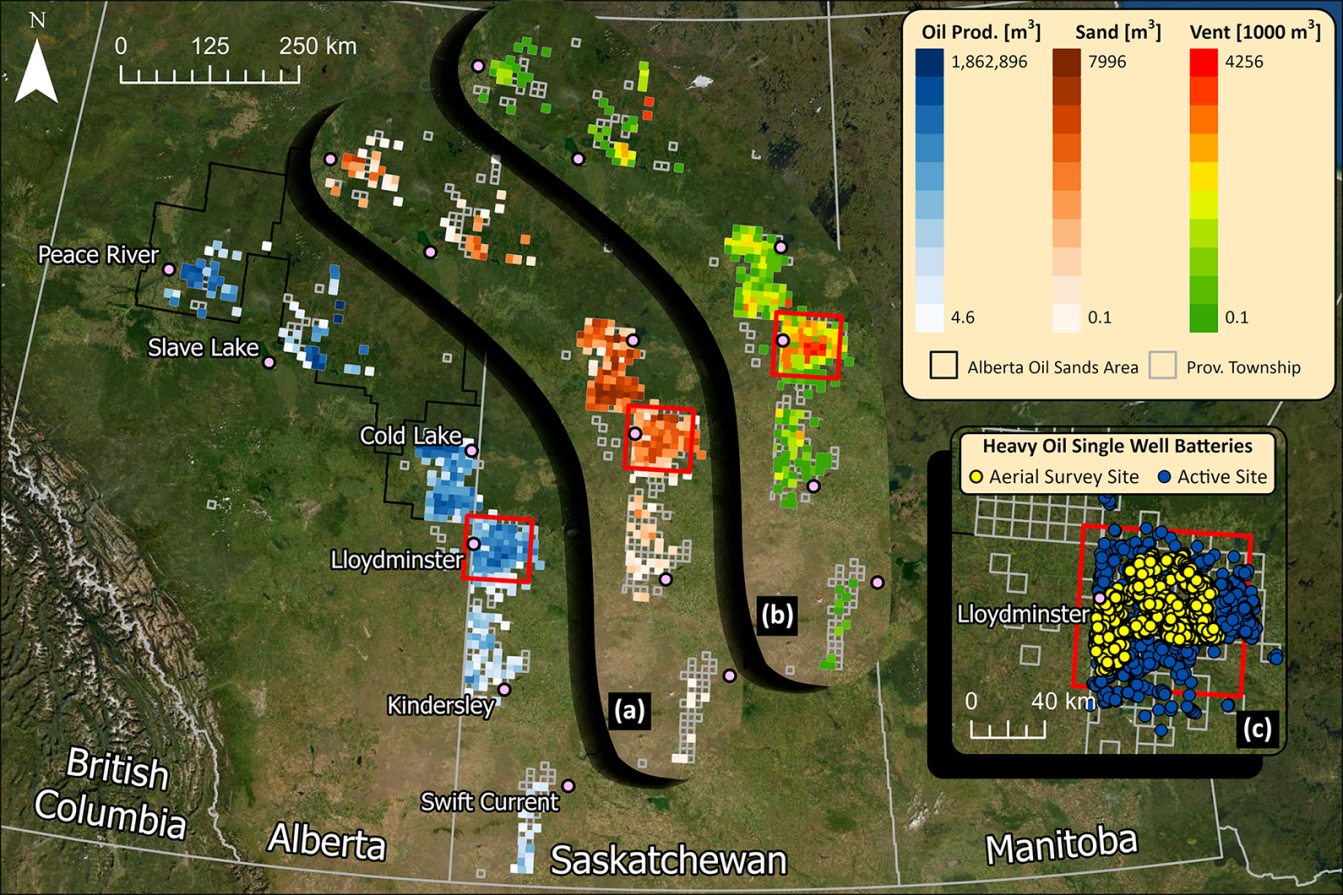
Map of the heavy oil belt (HOB) as gleaned from reported conventional cold heavy oil production volumes in 2021 by township in Alberta and Saskatchewan. Inset map (a) shows reported dispositions of sand, which is a marker for CHOPS (Cold Heavy Oil Production with Sand) facilities. Inset map (b) shows industry-reported vent volumes which illustrate how venting in Saskatchewan is dominated by CHOPS production near Lloydminster. Lower right inset (c) shows a close in view of active facilities and the 962 single-well CHOPS facilities included in the aerial survey data.

Canada’s Heavy Oil Belt (HOB), a region that extends through the provinces of Alberta and Saskatchewan centered on the town of Lloydminster (Figure 1), is responsible for 28% of conventional oil production (i.e., excluding mined and in situ oil sands) in both provinces. Within this belt, conventional cold heavy oil production (CHOP) proceeds without or with coproduction of sand (CHOPS). In the latter case, sand is intentionally coproduced with the oil to improve recovery.^6^ While this type of production is mostly unique to Canada’s heavy oil industry, its success has led to CHOPS trials in a number of other heavy oil deposits around the world including Alaska’s North Slope basin^7,8^ and China’s Jilin province^9^ and Henan oilfields.^10^ CHOPS has also been suggested for heavy oil deposits in Venezuela, but significant development has yet to occur.^11^

CHOP(S) production has historically been characterized by higher levels of reported methane emissions than conventional light or medium crude production,^12,13^ and several authors have highlighted the potential for significant under-reporting of methane venting. Using mass-balance flights covering 2291 CHOPS wells in Alberta in 2016, Johnson et al.^14^ found that actual methane emissions were approximately five times greater than reported. At the same time, Roscioli et al.^15^ performed on-site releases at five CHOPS facilities and used a tracer ratio method to show that measured venting exceeded reported levels at four out of five sites. More recently Mackay et al.^16^ aggregated multiple truck-based measurement campaigns to estimate that approximately 7% of the energy extracted in the heavy oil region surrounding Lloydminster was lost due to either intentional venting or fugitive emissions. Another truck-based study observed that methane was predominantly emitted from the wellhead casing or the well’s engine shed, with higher rates of tank top venting only observed at newer sites.^17^ Curiously, in a rare study focusing specifically on the Saskatchewan side of the border, methane emissions from the Lloydminster area were estimated to be lower than reported in inventories using downwind truck-based measurements.^18^ However, this result may be due to a conservative decision to limit attribution of detected methane enhancements to nearby sites within 400 m of a road while ignoring sites further upwind, as well as uncertainties in active facility counts at the time of measurements in September 2020 following COVID-19 impacts on production.

Discrepancies between observed and reported vent volumes are likely related to flaws in the methodology of estimating methane venting from produced oil volumes using an assumed Gas Oil Ratio (GOR). Challenges both in measuring and extrapolating GOR data (which under regulations may be measured as infrequently as once per year^19^ or once every three years^20^) have long been known especially for heavy oil wells.^21,22^ Peachey^21−23^ has reported significant variability in measured GOR values, with a general trend that measured gas volumes were much higher than reported.^23^ In particular, Peachey^22,23^ has suggested that heavy oil formations can contain pockets of associated gas that are not in solution in the oil, negating the premise that produced gas is linearly correlated to produced oil. Moreover, few CHOPS wells meter portions of produced gas used for on-site fuel versus vented to atmosphere, leading to further inaccuracies in reported data. Regulations based on these data risk being ineffective, and a better understanding of venting from CHOP(S) is essential for Canada to meet its methane reduction commitments.

Although CHOPS facilities are not directly tracked in publicly reported data, CHOPS activity in the HOB can be estimated considering reported sand dispositions (Figure 1a) which illustrates how CHOPS is most heavily concentrated in the Lloydminster area (red box). For the province of Saskatchewan specifically, where 31% of conventional oil production and 38% of industry-reported vented gas volumes come from the HOB, the concentration of CHOPS facilities near Lloydminster is strongly correlated with the highest levels of industry-reported vent volumes^24^ (Figure 1b). Given that the overwhelming conclusion from previous studies conducted in Alberta was that methane emissions were higher than reported with a single study looking at the Saskatchewan side suggesting the opposite, a better understanding of the characteristics and magnitudes of methane emissions in this region is required. This is the primary focus of the present study. Such data are essential for crafting effective regulations centered on available mitigation technologies which, when combined with the high density of CHOPS wells in this region, is thought to create an important opportunity for high-impact, comparatively low-cost methane mitigation.^12^

Leveraging a combination of large-scale aerial methane measurements using Bridger Photonics’ Gas Mapping Lidar (GML) with direct, on-site time-resolved methane vent rate measurements, the objectives of this work were to (i) quantify unsteady venting characteristics of active CHOPS sites, (ii) compare direct on-site measurements with aerial measurements and quantitative optical gas imaging (QOGI) estimates, (iii) collect source-resolved aerial methane emissions data for a large sample of CHOPS facilities, and (iv) contrast measured data with industry-reported vent volumes and relevant regulatory limits to better understand the limits of current regulations and opportunities for mitigation.

## 2 Gas Production and Venting during Cold Heavy Oil Production with Sand (CHOPS)

At a typical CHOPS well, a progressive cavity pump lifts a slurry of sand, oil, gas, and formation water to the surface. As the slurry is removed, so-called “wormholes” – technically channels of sand suspended in a mixture of oil, water, and small gas bubbles usually containing >90% methane^6,25^ – begin to propagate from the well into the formation. These bubbles create “foamy oil” and create a critical driving force as they grow and expand, which helps keep sand in suspension and promotes flow toward the well bore.^25,26^ Heavy oil deposits can also contain pockets of associated gas or gas caps beneath the cap rock;^23,25^ especially in the later phases of a well’s life, gas “slugging” can occur when this associated gas is accessed via wormholes and episodically brought to the well.^22,25^ At the wellbore, this gas tends to separate and enter the annular space between the well casing and well bore and is termed “annulus gas” or “casing gas”. As with most other well types, casing pressure must be relieved to avoid restricting oil production, and especially, to avoid pushing the liquid column down to the point where gas enters the pump and leads to cavitation and motor failure.^27^

**Figure 2:**
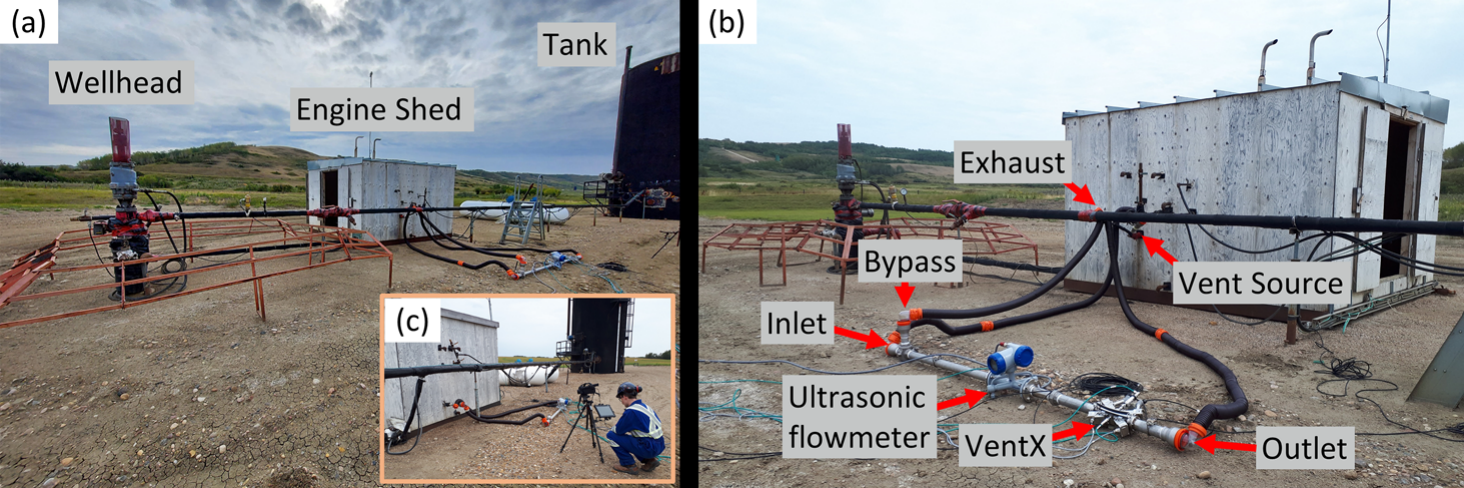
(a) Overview of a typical single-well CHOPS site showing the three main components: wellhead, engine shed, and storage tank. (b) Labeled photo of the ground-based instrumentation installed on the engine shed vent seen in (a). Measured gas has been routed back to the shed to exhaust adjacent to the original outlet. (c) QOGI measurement of an engine shed vent, simultaneous with the ultrasonic and VentX instruments (different site from (a) and (b)).

Referring to Figure 2a, casing gas may be vented directly at the wellhead or is often directed to an “engine shed”, where a portion may feed the engine that drives the pump’s hydraulics. Facilities like the one shown will often also have on-site tanks (“bullets”) of propane, which is used as a backup, supplement, or replacement fuel source for the engine. Excess or unused produced gas is then intentionally vented to the atmosphere from the shed. Any gas that remains in solution with the oil/water/sand slurry will be pumped into the storage tank. At a CHOPS site, the production storage tank acts as a simple vertical separator from which oil, sand, and water can be individually offloaded into dedicated trucks. These tanks are typically heated by a combustion heater to promote flow of the heavy oil. This combustion heater may also use a portion of the casing gas or draw from the on-site propane tanks. Any gas released from solution in the atmospheric pressure tank is vented directly to the atmosphere.

## 3 Methods

To maximize the sample size within the budgeted flight time, this study considered the region of Saskatchewan, Canada with the highest concentration of active, single-well CHOPS facilities. Within the contiguous region shown in Figure 1c, all sites containing at least one active single-well CHOPS site (i.e., active based on reported oil or gas production, flaring, venting, or fuel use volumes in the Petrinex reporting system^24^ at the time of planning during January-March 2021), and having sufficient resolution satellite imagery to precisely identify site boundaries within a few meters, were included in the aerial sample. Aerial measurements were completed using Bridger Photonics’ Gas Mapping LiDAR (GML) technology^28−31^ during August 2021. Due to changes in well status between the time of planning and measurements, as well as the presence of additional wells within some pads, the final sample included 827 active (at the time of measurement) and 135 inactive (“Shut-In”) single-well CHOPS sites for a total aerial sample of 962 single-well CHOPS facilities. Given that facilities will commonly switch back and forth between active and “shut-in” in the Petrinex reporting data as well as recent truck-based measurements^18^ suggesting inactive sites may be important emitters, the subset of 135 inactive (“Shut-In”) facilities was seen as particularly valuable.

In parallel with the aerial measurements, additional on-site, direct measurements of engine shed vents were completed at 11 facilities (the “ground sites”) as illustrated in Figure 2. Importantly, pursuant to the goals of measuring venting characteristics of emitting sites and collecting direct measurement data to compare with aerial measurements, the ground team preferentially chose these 11 sites because they were qualitatively observed to be emitting. By contrast, the aerial survey set was a random sample of all locatable active CHOPS sites within the contiguous region near Lloydminster.

Bridger Photonics’ GML is an aircraft-mounted LiDAR and camera system that leverages Wavelength Modulation Spectroscopy (WMS) to detect methane plumes between the plane and the ground and produce geolocated plume imagery at 1–2 m resolution. A detected plume’s path-integrated methane volume fraction and height above ground level is combined with wind speed data (typically from the NOAA High-Resolution Rapid Refresh (HRRR) model^32^) to infer a release rate. Using the probability of detection and uncertainty models of Conrad et al.^33^ derived through parallel controlled release studies in Saskatchewan, detection sensitivities during the present flights were 0.3–1.8 kg/h (at 50% probability of detection) with single- and multipass measurement uncertainties of −65/+141% and −41/+63%, respectively. These uncertainties are consistent with those found in tests by Bell et al.^34^ This detection limit is near the 1 kg/h threshold suggested as sufficient to capture all significant methane sources at upstream oil and gas facilities.^35^ Manual review of high-definition photography captured from the plane permitted detected emissions to be associated with specific emitting infrastructure on each site. Follow-up ground inspections at the 57 highest emitting sites in May 2022 validated these attributions.

For all 962 surveyed sites, initial flights typically consisted of two or more overlapping measurement passes as necessary to fully scan the site with the GML’s 94–134 m wide laser measurement swath (altitude dependent). Each site with detected emissions was reflown a second time on a subsequent day to rescreen detected sources and get a second measure of emissions, again with multiple passes where necessary. The average emission rate for each source was calculated by averaging single-pass emission rates for each flight day and then averaging over both flight days as in Johnson et al.^31^ Emission rates and uncertainties were computed using a Monte Carlo method leveraging the controlled release data and uncertainty models in Conrad et al.^33^

At the 11 ground sites, on-site measurements of engine shed vents were completed using three different technologies. The primary measurement tool was a pair of VentX meters–a WMS-based optical methane mass flux sensor^36,37^ that provides time-resolved (1 Hz) measurements of methane mass flow rate, methane fraction, and total gas flow rate in an intrinsically safe setup suitable for use in flammable gas environments. The VentX system deployed for this study has a methane mass flow uncertainty of ±0.40 kg/h at 95% confidence.^37^ At each of the 11 ground sites, a VentX meter was directly connected to the engine shed vent using a flexible hose to collect continuous time-resolved measurements of unsteady vent rates (see Figure 2b). A second length of hose routed the vent gas back to its original outlet location to avoid influencing any synchronous aerial measurements. VentX data were acquired for 3 to 21 h at each site depending on the duration required to characterize the flow based on observed trends in the real time data. At seven of the 11 sites, the VentX meter was recording flow rate data as the GML plane flew overhead. Asynchronous aerial measurements during reflights at these sites, or during both flights at the other four ground sites, were completed within 3 days prior to 6 days after the VentX measurement.

Additionally, at each site, a 2” ultrasonic flow meter (Khrone, Optisonic 7300 C/i-Ex) was installed in series. Although the ultrasonic meter could only measure total gas flow rate, it was useful to validate the total flow rate measurements of the VentX meter as further shown in the Supporting Information (SI, Section S1). Extractive gas samples were also collected from the VentX cell using a hand pump at 10 of the 11 sites and sent for gas chromatography (GC) analysis by a third-party laboratory. As further detailed in the SI (Section S2), measured methane fractions were similar in all cases after accounting for missing water vapor in the GC analysis, which considers dry gas only. Finally, when possible, simultaneous measurements of the engine shed vent rates were made using a QL320 quantitative optical gas imaging (QOGI) system.^38,39^ QOGI measurements were not recorded at sites where the camera’s field of view was obstructed by well infrastructure or where no suitably contrasting thermal background was available. The QOGI system consisted of a FLIR GFx320 camera (*f* = 23 mm lens) and the associated QL320 tablet with FLIR QL320 quantification software (v. 1.4.1). A response factor for pure methane was selected (0.297), which is consistent with the subsequently measured methane fractions of 86–93% as further discussed below.

## 4 Results and Discussion

### 4.1 Venting Characteristics of CHOPS Sites

**Figure 3:**
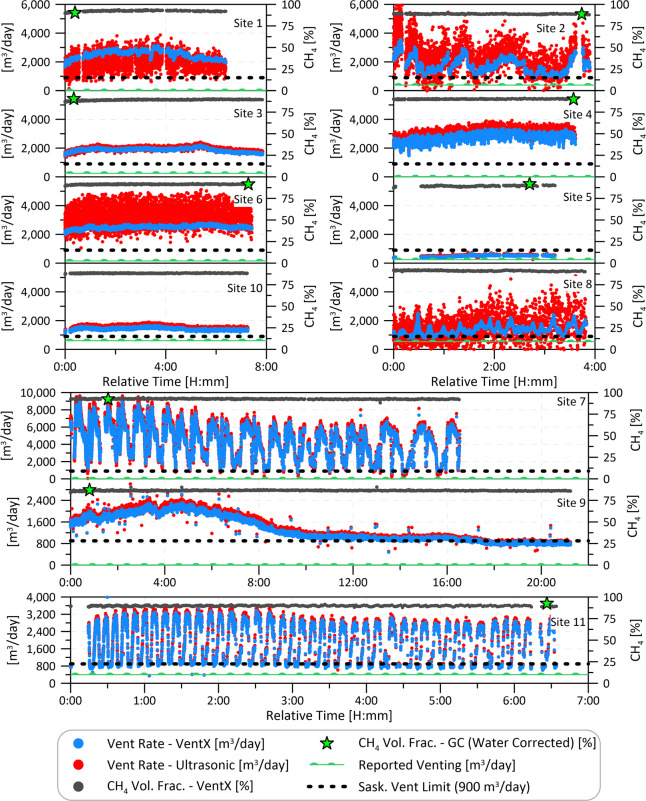
Measured time-resolved vent rates and methane volume fraction from engine shed vents at 11 CHOPS sites. Reported gas venting to Petrinex during August 2021 for each site is also shown (Sites 1, 4, 7, and 9 reported zero venting) as well as the regulated site vent limit of 900 m^3^/day. The methane volume fractions from the extractive gas samples, analyzed by GC and corrected for humidity, are shown as green stars. No gas sample was collected at Site 10; the sample at Site 8 was collected the following day.

Figure 3 plots the measured time-resolved total vent rate (whole gas) and methane volume fraction data from the engine shed vents at the 11 ground sites (See Figure S3 for equivalent methane mass flow rate data). As expected from CHOPS wells, the methane volume fraction was consistently high (86 to 93%) and varied by no more than ±1.5% at each site. By contrast, the time-resolved vent rates revealed a range of profiles. While some sites had a relatively constant vent rate (i.e., sites 1, 3, 4, 5, 6, 9, and 10), others varied by at least a factor of 3 in patterns that repeated multiple times over the measurement period (i.e., sites 2, 7, 8, and 11). This observed variability is a key factor in deciding on the most appropriate mitigation technology, where parameters such as maximum vent rates and turndown ratio must be considered. These results suggest that some sites may be easier to mitigate than others and that mitigation technologies with large turndown ratios will be required in some cases. However, an inquiry with one manufacturer of enclosed combustors suggests that the observed flow rate variability in Figure 3 is within the operating range of current systems so long as some minimal input pressure (≲2 psig) can be maintained. Because a pressure regulator is often installed shortly upstream of the engine shed vent outlet, presumably to maintain sufficient back pressure for the engine, this pressure requirement should already be met. Moreover, field notes from the ground team noted gas pressures as high as 15 psig (100 kPa) on visible pressure gauges.

The flow variability and range at several sites illustrate how field measurements of vent rates and hence GOR can be challenging, especially if using devices such as orifice meters which are prone to error in unsteady flows.^40^ Similarly, although the methane fractions are relatively steady, differences in gas composition can still present a challenge for techniques relying on thermal properties of the fluid (e.g., thermal mass flow meters) or fluid dynamic drag (e.g., turbine meters). Although the ultrasonic flow data closely track the time-varying VentX data at Sites 7 and 9 in particular, there is significant visible noise in the ultrasonic flow meter readings at Sites 1, 2, 6, and 8. This is potentially related to damping of the acoustic signal from other species in the flow such as carbon dioxide^41,42^ or from acoustic noise from the upstream engine.^43^ The VentX instrument appears well-suited to this application.

Notwithstanding that these 11 sites were preferentially chosen due to observed venting, measured vent rates at all but one site (Site 5) were consistently above the Saskatchewan site-total venting limit of 900 m^3^/day.^19^ Moreover, the measured rates at all 11 sites exceeded corresponding reported volumes during the month of the measurements, with four sites (1, 4, 7, and 9) reporting no vented gas despite measured mean gas flow rates of ∼1,300–4,300 m^3^/day. Additionally, only the engine shed vents were measured; any potential contributions from other vent sources (e.g., storage tanks or well-head vents) were not considered.

### 4.2 Comparison of Aerial and Ground-Based Measurements

**Figure 4:**
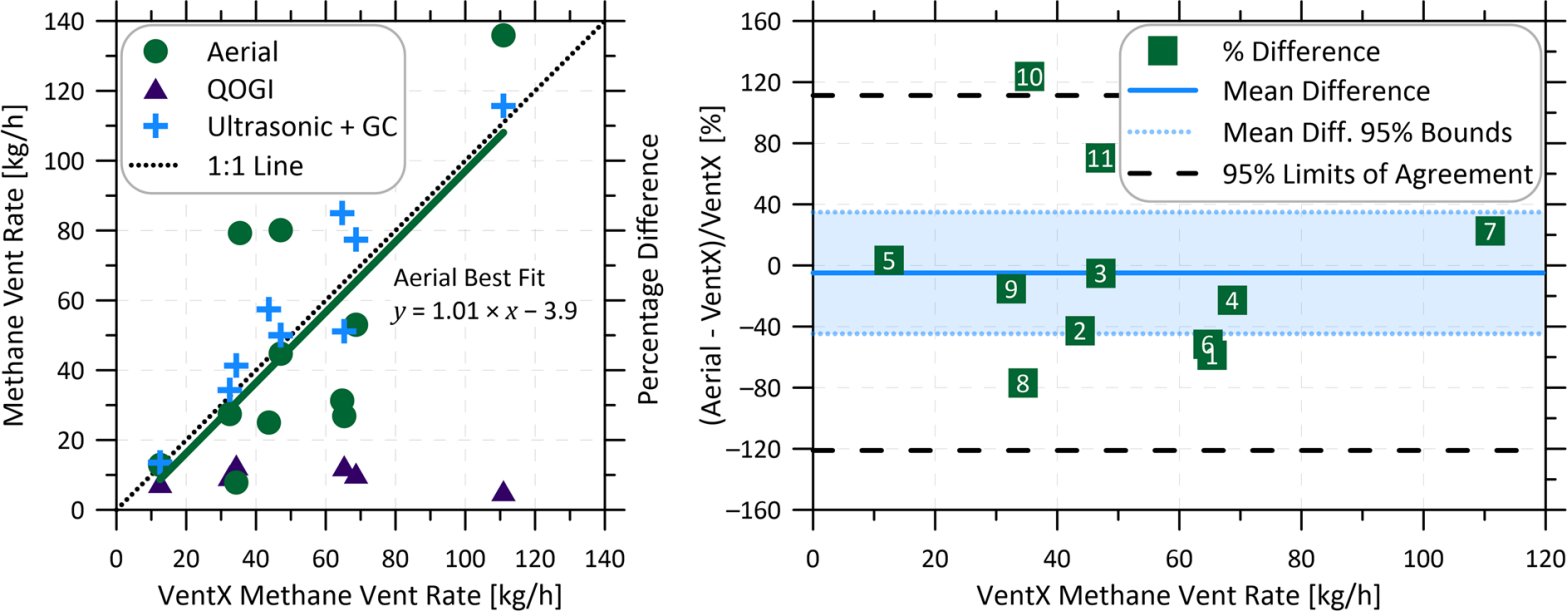
(a) Comparison of engine shed methane vent rates as measured by the aerial GML, the QOGI camera, and the ultrasonic flow meter (using the gas sample methane results corrected for water saturation) versus the VentX sensor mean vent rate. (b) Bland-Altman plot of the percentage difference between the aerial and VentX measurements (labels on the data points indicate site numbers from Figure 3). The 95% limits of agreement (long black dashes) are expected to contain 95% of differences between the two instruments.

Figure 4a compares the average methane vent rates measured via the aerial GML, the ultrasonic flowmeter combined with GC samples, and the QOGI camera with the average rates recorded by the VentX meter for the engine shed vents at the 11 ground sites. Tabulated data are included in the SI (Table S2 and Figure S4). The 1:1 line shows the excellent correlation between the ultrasonic and VentX meter, where the scatter is attributable to the noise in the ultrasonic readings at some sites (see SI) and the need to humidity correct the GC measurements as part of calculating methane flow from the ultrasonic flow measurements. More importantly, although the sample size of 11 is small, the correlation between the GML and VentX results is nearly perfect, as seen in the included linear fit result. This observed correlation holds despite multiple sites having variable vent rates, and the time difference between the GML and VentX measurements stretching to 6 days in some cases. This important result suggests that, on average, the GML technology provides accurate estimates of CHOPS vent rates.

To investigate this further and to further support the use of the aerial technique in quantifying methane vents on a large scale, Figure 4b shows a Bland–Altman (B–A) plot of the percentage difference between the aerial and VentX measurements versus the VentX vent rate. B–A plots are a simple graphical analysis tool used to compare the performance of two instruments with the same measurement task.^44,45^ The B–A plot indicates that GML may have a slight negative bias compared to the VentX, with an average measurement difference of −4.9% (i.e., conservatively low). However, the sample is small such that the 95% confidence bounds on this mean difference span from +35% to −45%. Encouragingly, referring to the data point labels corresponding to the site identifications in Figure 3, there is no obvious difference in agreement linked to the variability of the vent rate. For any one site, the 95% confidence limits of agreement (long black dashes) indicate that 95% of differences between the aerial and VentX method will fall between 111% and −121%. While this accuracy is slightly worse than the range predicted by Conrad et al.^33^ using data from steady controlled releases, it is not unexpected due to the observed emission rate variability and the inclusion of asynchronous measurements. Overall, these results indicate that, on average, the aerial GML will give a good estimate of the vent rates measured by the VentX and that the two techniques are highly complementary: while the GML enables large-sample measurements at the inventory scale providing insights into how venting characteristics are distributed across a large population, the VentX provides accurate, time-resolved measurements for site-level reporting and assessment of site-specific mitigation solutions.

The QOGI camera on the other hand was a complete failure, showing no proportional relationship between reported flow rate and actual flow rates as verified by the VentX and ultrasonic flowmeters. The QOGI technology is thus unable to accurately quantify these high vent rates. According to the manufacturer of the QOGI camera, the system is calibrated using propane from 0.1 to 30 LPM and can be “safely” extended to 300 LPM for methane but that plumes with “high exit velocities can be underestimated”.^38^ This extrapolated upper limit corresponds to ∼432 m^3^/day or 12.2 kg/h of methane, which is below the flow range of most sites in Figure 3. While it is understandable that the QOGI may not perform well beyond its intended range, from a practical point of view it is concerning that in the present tests the system output rates in the range of 5.5–12.9 kg/h without any obvious indication to the user that these may not be accurate.

### 4.3 Measured versus Reported Venting at CHOPS Sites

**Figure 5:**
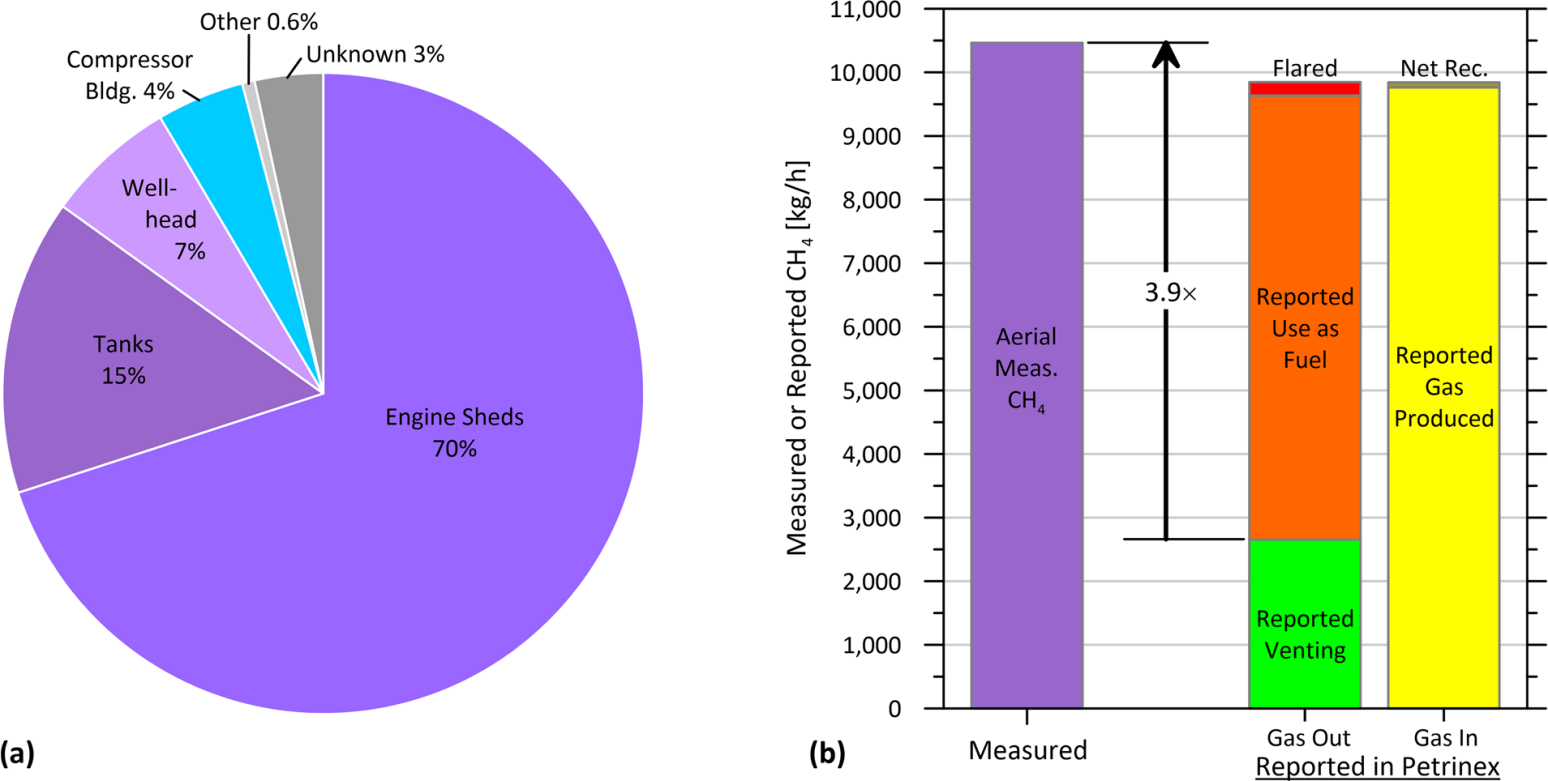
(a) Relative contributions of specific sources to total measured methane emissions in the aerial survey of 962 single-well CHOPS sites. (b) Comparison of the measured total methane emission rate (purple) with monthly gas volumes reported in Petrinex, compiled as the total reported gas out and total gas in at the same sample of 962 sites during the month of measurements.

The breakdown of individual sources as a percentage of total methane detected during the aerial survey is plotted in Figure 5a. Engine shed vents are by far the dominant source, accounting for 70% of all detected methane emissions. Tank vents were the next biggest source at 15% of total emissions, with direct venting of gas at the wellhead accounting for a further 7%. Although 4% of emissions were attributed to compressors, follow-up investigations suggest these were likely vented sources rather than combustion emissions, originating either from a bypass vent line at the compressor inlet regulator or from venting from associated pneumatic equipment and pumps. Thus, ∼81% of methane emissions from CHOPS wells is attributable to venting of high-methane content casing gas from ground-level sources. A recent technoeconomic analysis of flaring and venting mitigation options in neighboring Alberta suggests there are several potential options for cost-effectively eliminating these sources.^12^ Similarly, although tank venting would be more challenging to reduce due to the location of the vent and the possibility of variable gas composition and associated oxygen ingress,^46,47^ the widespread deployment of vapor recovery units at heavy oil sites in the Peace River area of Alberta suggests this is feasible.^17^

Figure 5b shows that the aggregate measured methane emissions of 10,466 kg/h of the 962 CHOPS facilities in the aerial sample are 3.9 times greater than the total reported venting at these same sites (calculated as 2655 kg/h, assuming the total reported vent gas volume of 3,103,400 m^3^/mo has an average methane fraction of 93.8% consistent with Saskatchewan government emissions factors^48^). Moreover, this measured emission rate approximately equals the total reported gas production and receipts of 9844 kg/h. This suggests both that gas being reported as fuel use is being vented, and that overall gas production is underestimated. Presumably this underestimation is directly related to uncertainties associated with GOR measurements. While surprising, this is supported by Figure 5b, which shows there is no net conservation of gas from these sites. Specifically, among these sites, the total reported gas outputs of venting, fuel use, and flaring exceed the total reported gas production with a small amount of gas being delivered into the sample set from elsewhere. Thus, there are no net dispositions of gas from these sites into the external pipeline network and hence no incentive or need for accurate gas measurements. This significant underestimation is similar to the mismatch between measured and reported data seen at CHOPS sites in Alberta in 2016, measured using a different aerial technology.^14^ This persistent mis-reporting of venting volumes and the resulting inaccuracies in bottom-up GHG inventories pose considerable risk to reduction targets and the creation of efficient regulations, including those currently under development in Canada. Future regulations will need to enforce minimum standards for measurement if emissions are to be verifiably reduced.

Of the 827 active sites (at the time of measurement) in the sample, 76% (626) were emitting detectable methane by GML for a population-average methane emission rate (including nonemitting/undetected active sites) of 12.5 kg/h/site (12.0–13.1 kg/h/site at 95% confidence). By contrast only 10% (13) of the 135 shut-in sites were observed to be emitting, with a population-average emission rate of 0.83 kg/h/site (0.64–1.13 kg/h/site at 95% confidence). Scaling these to the estimated 1917 active and 1314 inactive single-well CHOPS facilities in Saskatchewan suggests total methane emissions of approximately 220.0 kt/yr (198.4–243.6 kt/yr at 95% confidence considering effects of aerial measurement uncertainty, sample size, and finite population^49^) comprising 210.4 (190.1–232.4) kt/yr from active sites and 9.6 (3.2–18.9) kt/yr from inactive sites. Thus, although inactive sites contribute measurable methane, they represent only 4.4% of emissions. Notably, this is ten times less than suggested by Vogt et al.^18^ for truck-based measurements near Lloydminster, although a close inspection of their results reveals several instances in which emissions appear to have been attributed to inactive sites close to the road while the aerial GML detected emissions from neighboring active sites further upwind. More importantly, the presently calculated 220.0 kt/yr of methane emissions from CHOPS sites alone is approximately 61% of the estimated 360 kt of fugitive methane emissions for the entire Saskatchewan upstream oil and gas sector in the latest federal inventory.^50^

In Saskatchewan, directive PNG036 from the Ministry of Energy and Resources sets the per-site vent limit at 900 m^3^/day (total gas, equivalent to 23.9 kg/h at 93.8% CH_4_)—any site which exceeds this limit must conserve and/or flare the associated gas.^51^ However, 14% of sites in the survey appear to be exceeding this limit (See Figure S5 of the SI). Moreover, these sites were responsible for 61% of emitted methane, a clear indication that current regulations have gaps. Relative to the much stronger regulations in place in neighboring Alberta,^52^ the present results are even more stark. The measured site average emission rate of 10.9 kg/h for the present sample falls just below the Alberta overall methane vent gas (OVG) limit (12.3 kg/h) and is 8.3 times greater than the Alberta fleet average limit (1.31 kg/h), described in the SI. Indeed, 79% of methane emissions are from sites emitting greater volumes than the Alberta OVG. These results suggest that stronger regulations will be required to meet methane reduction targets, and, in particular, illustrate the need for independent monitoring, reporting, and verification (MRV).^53,54^

### 4.4 Implications

**Figure 6:**
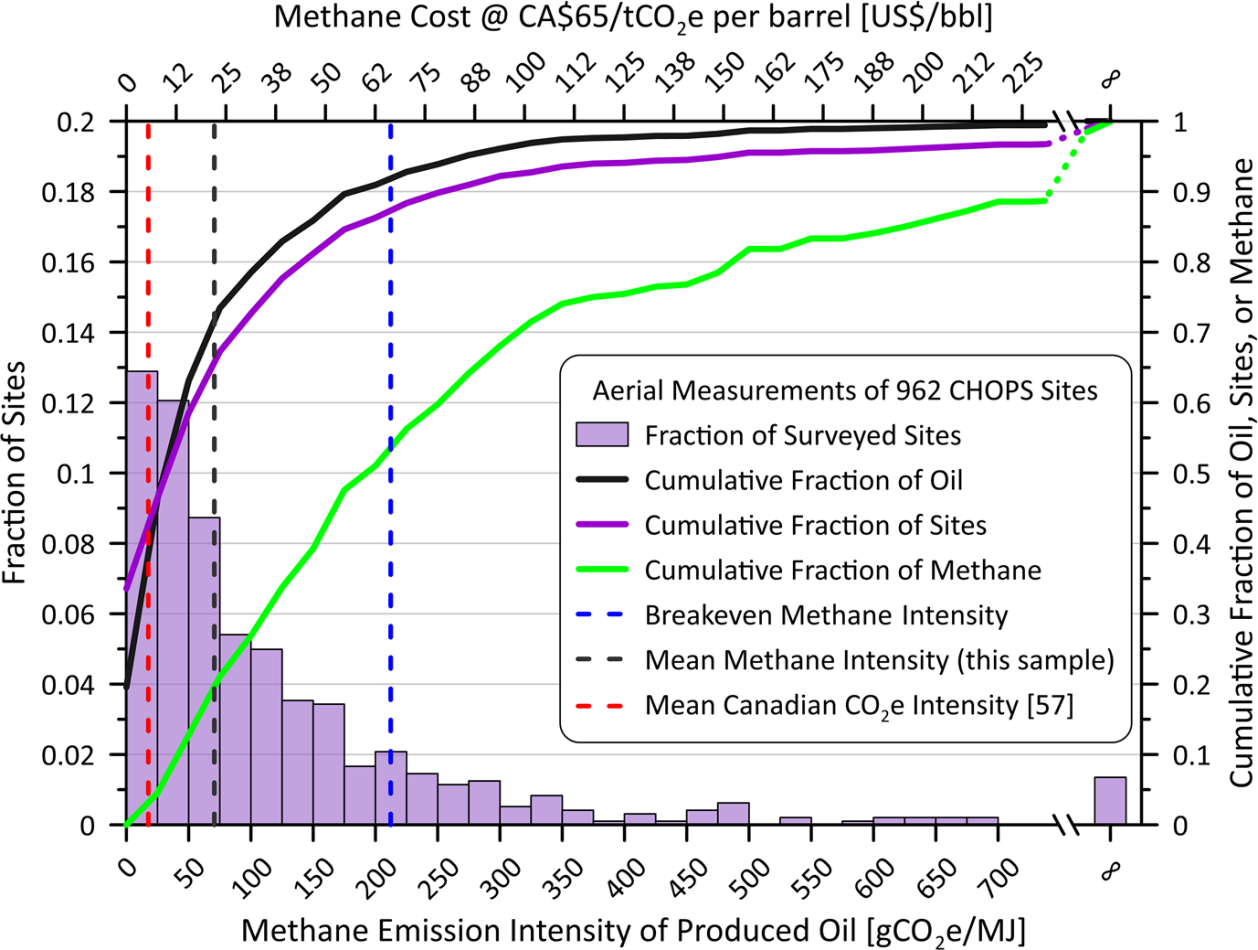
Measured methane emission intensity of the oil produced at CHOPS sites in Saskatchewan. The top horizontal axis indicates the methane cost per barrel of oil, based on the current Canadian carbon price of CA$65/tCO_2_e. The measured average *methane* intensity, 70.6 gCO_2_e/MJ, is displayed by a dashed black line, while the dashed red line is the Canadian average *carbon* intensity reported by Masnadi et al.,^57^ and the dashed blue line is the breakeven price using the reference Western Canadian Select oil price of US$66.38/bbl.

Figure 6 recasts the measured site-level methane emissions to consider methane intensity of the oil produced during July–September 2021 (i.e., the month before, during, and after the field measurements). The measured methane emission rates (in kg/h) were conservatively assumed to apply only during reported operating hours, ignoring potential contributions from inactive facilities.^18^ An energy density of heavy oil of 40.9 GJ/m^3^ was assumed,^55^ and a methane global warming potential (GWP) of 25 was used to convert to grams of CO_2_e. Although the current 20-year GWP value of 82.5^56^ would be the correct value to use for discussions of near-term 2030–2050 policy targets, the value of 25 is still commonly used in carbon pricing calculations.

The oil-production weighted, mean measured methane intensity of oil produced by this sample of CHOPS facilities is 70.6 gCO_2_e/MJ (black dashed line on Figure 6). Critically, this derived methane emission intensity does not account for carbon dioxide emissions nor emissions from other parts of the well’s life-cycle. Even so, this value is still four times greater than the estimated mean total life-cycle *carbon* intensity of Canadian oil of 17.6 gCO_2_e/MJ, derived by Masnadi et al.^57^ (dashed red line on Figure 6), highlighting the environmental burden of CHOPS production in Saskatchewan. Moreover, approximately half of the methane in this sample is associated with only 10% of the produced oil. Indeed, in 13 instances the GML system measured methane vent rates from sites with no reported oil production during the three months considered, resulting in infinite methane emission intensity as noted at the end of the distribution.

This observation raises the concept of “marginal wells”, which denotes wells with relatively low production yet potentially high emissions. Traditionally, a marginal well has been defined as having a production rate equal to or less than 15 barrels of oil per day.^58,59^ However, this classification is not well-suited for single-well CHOPS sites, where 72% of sites in our aerial sample fall below this level and the mean production rate across all 962 sites is 14.5 bbl/day. Instead, we suggest an alternative definition of marginal wells based on the assumed cost of carbon emissions relative to the value of the produced oil.

Figure 6 includes a secondary horizontal axis showing the cost of the emitted methane per barrel of oil using the current Canadian carbon price of CA$65/tCO_2_e (equivalent to ∼US$48/tCO_2_e). Although this price does not currently apply to methane venting in Canada, the new U.S. Inflation Reduction Act^60^ has notably introduced a charge on applicable oil and gas sector methane emissions with an initial rate of US$900/tCH_4_ that converts to US$36/tCO_2_e (CA$49/tCO_2_e) and will rise to US$60/tCO_2_e (CA$81.67/tCO_2_e) by 2026.^60^ “Environmentally marginal wells” could thus be defined relative to the breakeven emission intensity, where the theoretical or applied cost of methane emissions equals the value of the produced oil. Using the current Canadian carbon price, this breakeven point was calculated assuming the October 2022 marketed heavy oil price of US$66.38/bbl (Western Canadian Select, WCS)^61^ as shown in the figure by the dashed blue line (212.2 gCO_2_e/MJ). This price ignores further reductions in the value of raw product at the production site relative to the WCS blend and all operating costs. Notwithstanding this favorable pricing and ignoring any carbon dioxide emissions from the engine driving the well, 47% of all emitted methane was released from marginal wells with zero or negative value. As shown in Figure S6, this fraction will only increase as the Canadian carbon price increases to CA$170/tCO_2_e by 2030^62^ (∼US$125.80/tCO_2_e).

However, previous technoeconomic analysis suggests methane emissions can be readily reduced or eliminated via a range of commercially available mitigation solutions, including compression of gas into pipelines for sale, combustion in auxiliary burners, or combustion in stand-alone combustors.^12^ Considering only the combustor option, which is generally simplest to implement but does not offer any revenue potential through produced gas, Figure S7a shows that applying the current carbon price of CA$65/tCO_2_e could eliminate 97% of methane with simple payback periods of less than 2 years. At CA$170/tCO_2_e, 99% of methane could be eliminated with a payback period of less than 1 year. Notably, relative to the value of the produced oil, Figure S7b shows that the expected costs of methane mitigation represent less than 12 months of produced oil value at 98% of sites, which comprise 98% of the emitted methane from producing sites in the sample. This suggests that with accurate measurement and application of current carbon price targets to methane emissions (either directly or as a guide to justify mitigation costs), reductions of 75% or greater should be readily achieved.

## Supporting Information Available

Additional graphs, discussion, and references (PDF)
Underlying data tables (XLSX)
