# Supplementary material for: Methane Venting at Cold Heavy Oil Production with Sand (CHOPS) Facilities Is Significantly Underreported and Led by High-Emitting Wells with Low or Negative Value
Source: Environ Sci Technol. 2023 Feb 6;57(8):3021–30. doi: 10.1021/acs.est.2c06255 (PMC9979599; doi:10.1021/acs.est.2c06255)
Supplement: Supplementary file 1 — es2c06255_si_001.pdf [file es2c06255_si_001.pdf]

Supporting Information

Methane Venting at Cold Heavy Oil Production with Sand (CHOPS) Facilities is Significantly Underreported and led by High-Emitting Wells with Low or Negative Value

Simon A. Festa-Bianchet, David R. Tyner, Scott P. Seymour, Matthew R. Johnson*

Energy & Emissions Research Laboratory,
Department of Mechanical and Aerospace Engineering,
Carleton University, Ottawa, ON, Canada, K1S 5B6

*Corresponding author: Matthew.Johnson@carleton.ca

File contains 11 pages, 7 figures, 5 tables

TABLE OF CONTENTS

S1 Comparison of VentX and Ultrasonic Total Flow Measurements S2

S2 Analysis of Collected Gas Samples and Correction for Humidity S2

S3 VentX Methane Mass Flow Results S4

S4 Engine Shed Average Methane Vent Rates S5

S5 Distribution of CHOPS Methane Emission Rates S6

S6 Marginal Well Fractions and Potential Mitigation Considering Current and Future Prices on Carbon S7

S7 References S9

S8 Underlying Data Tables S11

S1 Comparison of VentX and Ultrasonic Total Flow Measurements

As described in Sections 3 and 4.1–4.2 of the main text, an ultrasonic flow meter was installed in series with the VentX meter at all eleven ground sites to confirm and validate the latter's flow rate measurement in the field. Figure S1a compares the total gas flow rate measurements of these two meters. As discussed in Section 4.1 and shown in Figure 3, the ultrasonic flow meter signals were visibly noisy at Sites 1, 2, 6, and 8. This is evident as added scatter on the left of Figure S1a below. Although the reason for the noise in the ultrasonic signals at these sites is not known, it is speculated that it may be related to damping of the acoustic signal from other species in the flow such as carbon dioxide[1,2] or from acoustic noise from the upstream engine[3]. Ignoring the data from these sites, the remaining data in Figure S1b reveal a close correspondence and a clear linear trend (slope of 1.07) between the total flow rate measured by ultrasonic and VentX meters.

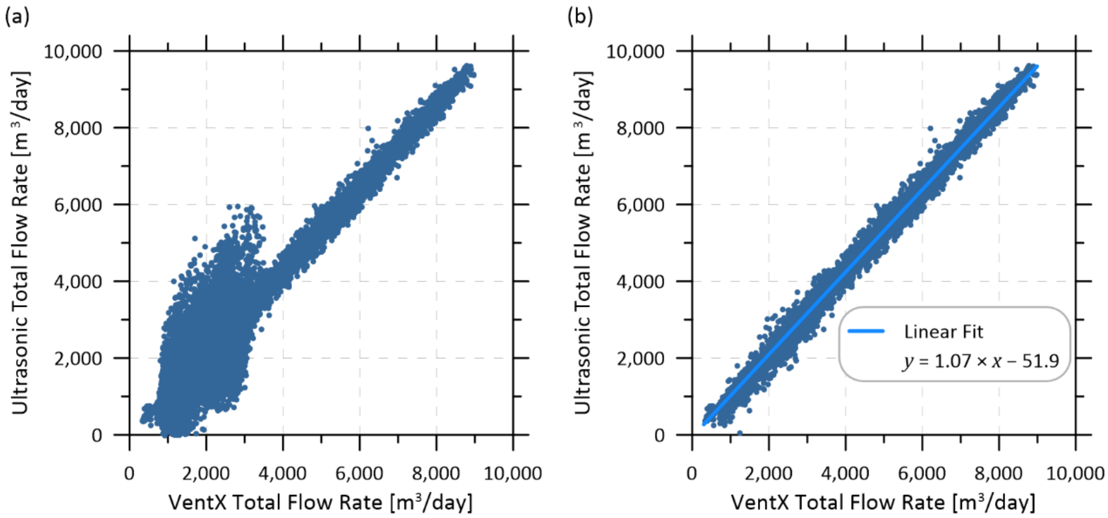

Figure S1: (a) Total gas flow rate as measured by the VentX compared to the ultrasonic flow meter for all eleven ground sites. (b) Total gas flow rate as measured by the VentX compared to the ultrasonic flow meter for select sites (3, 4, 5, 7, 9, 10, and 11).

S2 Analysis of Collected Gas Samples and Correction for Humidity

Extractive gas samples were collected for separate laboratory gas chromatograph (GC) analysis at ten of the eleven ground sites. The samples were collected from a port on the VentX meter and permit comparison with the time-resolved methane fractions measured by the VentX (See Figure 3 of the main text) as tabulated in Table S1. Critically, standard gas sample collection and lab-based GC analyses like these do not preserve liquid in the sample and any water vapour is ignored by the GC instrument. Sample analyses are thus reported on a dry basis and must be humidity-corrected prior to comparing with any direct measurements acquired in the field that measure the actual gas composition as is in the sample (i.e, including the influence of water vapour).

Given that produced annulus gas at CHOPS wells is expected to be saturated with water[4,5], it is possible to correct dry gas analyses by adding back the expected water mass corresponding to saturation conditions for the temperature and pressure at the time of sample collection. Absolute water content was estimated using

the charts provided by Campbell[6] for sweet natural gas, using the measured temperature and pressure from the VentX sensor. Figure S2 plots the raw GC-reported methane fractions in the samples (open circles) and the humidity corrected values (filled circles) versus the VentX reported methane fractions. Tabulated data for the figure are provided in Table S1. The results agree well within the uncertainties of this correction and serve as a useful validation check on the VentX methane measurements. These humidity-corrected GC data were used to calculate methane emission rates from the ultrasonic meter data as shown in Figure 4 of the main text.

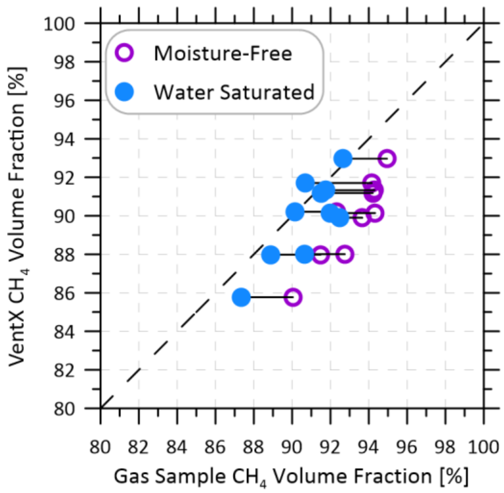

Figure S2: Methane volume fraction reported by the VentX sensor versus laboratory methane volume fraction from extracted gas samples for 10 of the 11 sites. The purple open circles indicate the moisture-free methane volume fraction, while the filled blue dots are the calculated corrected methane volume fraction for a water-saturated gas.

Table S1: Dry gas methane volume fractions from the gas chromatograph (GC) analysis of the collected samples, sample point temperature and pressure recorded by the VentX meter, estimated saturated water content of sweet natural gas from Campbell [6] (at 100 kPa and 15°C), and humidity-corrected GC methane fractions assuming water saturation in the samples. Directly measured methane fractions from the VentX meter included for comparison are the instantaneous measurements at the start time of the sample extraction.

Site	Raw GC CH_4 [%]	Temperature at sample point [°C]	Pressure at sample point [kPa]	Saturated Water Content [kg/10^6m^3]	Humidity-Corrected GC CH_4 [%]	VentX CH_4 [%]
**1**	94.2	29.5	93.1	28,750	90.7	91.7
**2**	91.5	24.5	94.1	22,000	88.9	88.0
**3**	92.8	21.0	93.8	17,500	90.6	88.0
**4**	92.3	21.5	93.8	18,000	90.2	90.2
**5**	94.3	22.5	93.1	19,100	92.0	90.1
**6**	94.3	24.0	93.8	20,900	91.8	91.3
**7**	95.0	22.0	93.1	18,800	92.6	93.0
**8**	94.2	25.0	93.8	22,000	91.5	91.2*
**9**	90.0	26.0	93.1	23,250	87.3	85.8
**10**	N/A	N/A	N/A	N/A	N/A	N/A
**11**	93.7	11.5	93.1	9,600	92.5	89.9

*The GC sample was taken the following day after the VentX measurements; the VentX value shown is the average during the previous day.

S3 VentX Methane Mass Flow Results

Figure S3 displays the continuous methane mass flow measurements of the VentX meter for the eleven ground site engine shed vents. These data were calculated by combining the instrument's simultaneous measurements of flow rate, methane volume fraction (See Figure 3 of Section 4.1), temperature, and pressure. The measurement start time at each site is included in each sub-plot.

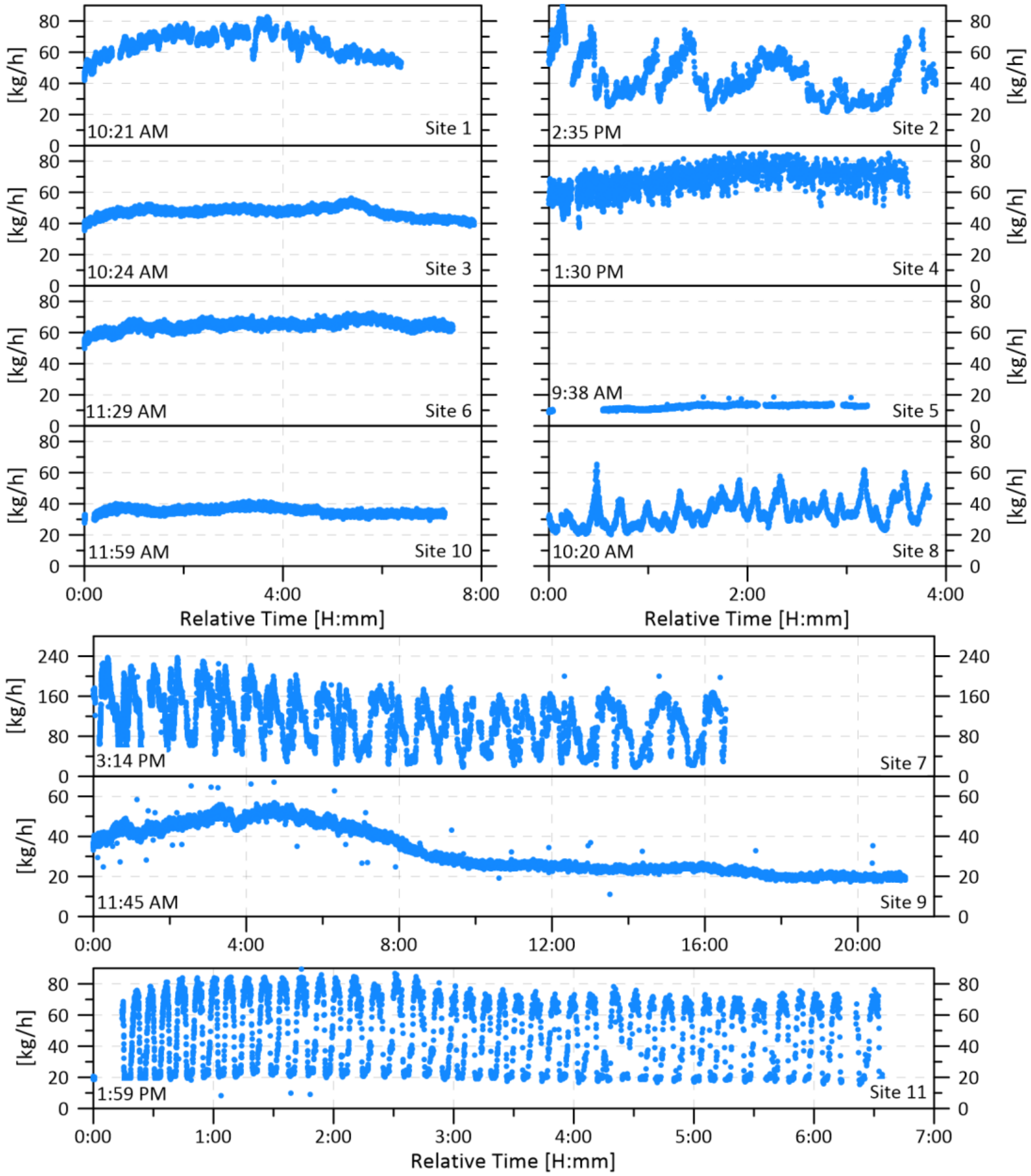

Figure S3: Measured time-resolved methane mass flow rates from the engine shed vents at eleven CHOPS sites, obtained using a VentX meter. The measurement start time at each site is included in the lower left corner of each subplot.

Although key oil and gas methane sources such as liquid storage tanks can be influenced by diurnal temperature changes that affect "breathing losses" from the vapour head space in the tank[7,8], such effects are not expected in casing gas venting at CHOPS sites (via the engine shed in the present case) since the

gas flows directly from the deposit (at a depth of 350–1000 m)[4] through the well's casing and production of the sand/oil/gas slurry is driven by a progressive cavity pump that tends to run continuously at constant speed. Indeed, closer inspection of the available data in Figure S3 / Figure 3 show no clear trends with time of day. Most sites (i.e., 2, 3, 5, 6, 8, 10, 11) are notably steady in their average; Site 4 shows a general slow increase through the afternoon; Site 7 shows the opposite; and Sites 1 and 9 show a modest peak between ~2–4pm. Similarly, while some other studies have noted potential for increased daytime emissions driven by manual operations by service personnel during working hours[9,10], there were no such operations at any of the sites in Figure S3 / Figure 3 during the on-site measurements.

S4 Engine Shed Average Methane Vent Rates

Figure 4 in Section 4.2 presented the average methane vent rate for the eleven engine shed vents which were measured by both the ground instruments and the aerial GML. These same data are provided in Table S2 and reproduced as a bar chart (Figure S4) to allow for direct comparisons among measurement methods at each site. Additionally, the industry-reported venting levels from Petrinex during the month of the measurements are included, where the methane emissions values have been calculated assuming a methane fraction of 93.8%. As explained in the manuscript, the GML results are averages of vent rates recorded over at minimum two separate flight days, 4 to 5 days apart. In some cases (Sites 1, 2, and 11), all flights occurred outside of the measurement period of the ground instruments. The GML values for the other eight sites include at least one flight day which was synchronous with the ground measurements. Missing QOGI measurements (Sites 2, 3, 6, 10, and 11) were due to either a poor thermal contrast between the gas plume and the site background, or obstructing site infrastructure that prevented adequate plume formation and imaging. Site 10 does not have an ultrasonic mass flow rate due to a lack of available extractive sample for methane fraction. The methane emission intensity, calculated using the VentX mean methane vent rate, is also included to facilitate comparison with Figure 6 of the main manuscript.

Table S2: Average methane vent rates for the engine shed vents at the eleven ground sites and industry reported venting for the site during the month of the measurements (calculated assuming a methane volume fraction of 93.8%).

Site	VentX [kg/h]	Ultrasonic + GC [kg/h]	Aerial [kg/h]	QOGI [kg/h]	Reported [kg/h] †	Methane Intensity of Produced Oil‡ [gCO_2e/MJ]
1*	65.3	52.1	26.8	12.7	0	541.7
2*	43.7	58.5	25.0	N/A	10.0	139.7
3	47.1	50.9	44.7	N/A	6.4	187.8
4	68.7	78.8	53.0	10.6	0	887.8
5	12.5	13.8	12.9	7.9	6.3	131.5
6	64.7	86.5	31.3	N/A	3.9	429.4
7	110.9	117.9	135.9	5.5	0	415.0
8	34.4	42.1	7.9	12.9	14.0	271.8
9	32.5	34.9	27.5	9.8	0	274.9
10	35.5	N/A	79.3	N/A	15.7	529.3
11*	47.1	51.0	80.1	N/A	10.4	321.8
Mean	51.1	58.7	47.7	9.9	6.1	375.5

*All aerial measurement passes were completed either before or after the ground measurements.

† Calculated from reported whole-gas data assuming a methane fraction of 93.8%.

‡ Calculated using the VentX mean vent rate, a CH_4 global warming potential of 25, and an oil energy density of 40.9 GJ/m^3.

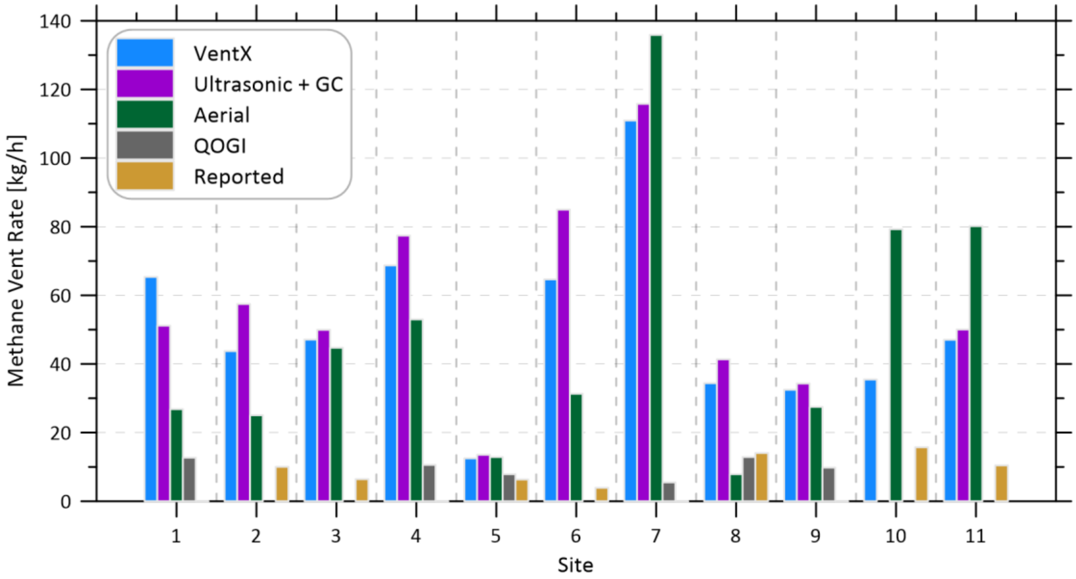

Figure S4: Comparison of average methane vent rates for engine shed vents using a VentX meter, ultrasonic flow meter and GC methane volume fraction, Aerial GML, and QOGI with reported site-wide venting in Petrinex [11] for the month of August 2021 (same month as measurements). Sites 1, 4, 7, 9 reported zero venting. QOGI measurements were not completed at Sites 2, 3, 6, 10, and 11.

S5 Distribution of CHOPS Methane Emission Rates

Figure S5 aggregates measured methane emissions by site to permit comparisons with relevant regulatory limits. Noting the broken scale on the horizontal axis, the distribution of site emissions is strongly right-skewed. As discussed in Section 4.3 of the manuscript, in Saskatchewan there is a regulated per-site venting limit of 900 m^3/day (total gas, equivalent to 23.9 kg/h at 93.8% CH_4) – any site which exceeds this limit must conserve and/or flare the associated gas[12]. The cumulative distributions in Figure S5 show that 14% of sites in the survey appear to be exceeding this limit and these sites were responsible for 61% of emitted methane. This points to a clear gap in enforcement of current regulations. This graph also highlights the weakness of Saskatchewan regulations relative to those in neighboring Alberta, which has two vent limits relevant to CHOPS sites. First, all sites must adhere to an overall methane vent gas (OVG) limit of 9,000 kg/month (12.3 kg/h), while "crude bitumen batteries" specifically (i.e., CHOPS facilities) must have a "fleet average" vent rate (i.e., average site vented emissions by company) of not more than 1,500 m^3/month (1.31 kg/h at 94% CH_4)[13]. Notably, the presently measured site *average* emission rate of 10.9 kg/h for all CHOPS sites in Saskatchewan sample falls just below the Alberta OVG limit and is 8.3 times greater than the Alberta fleet average limit.

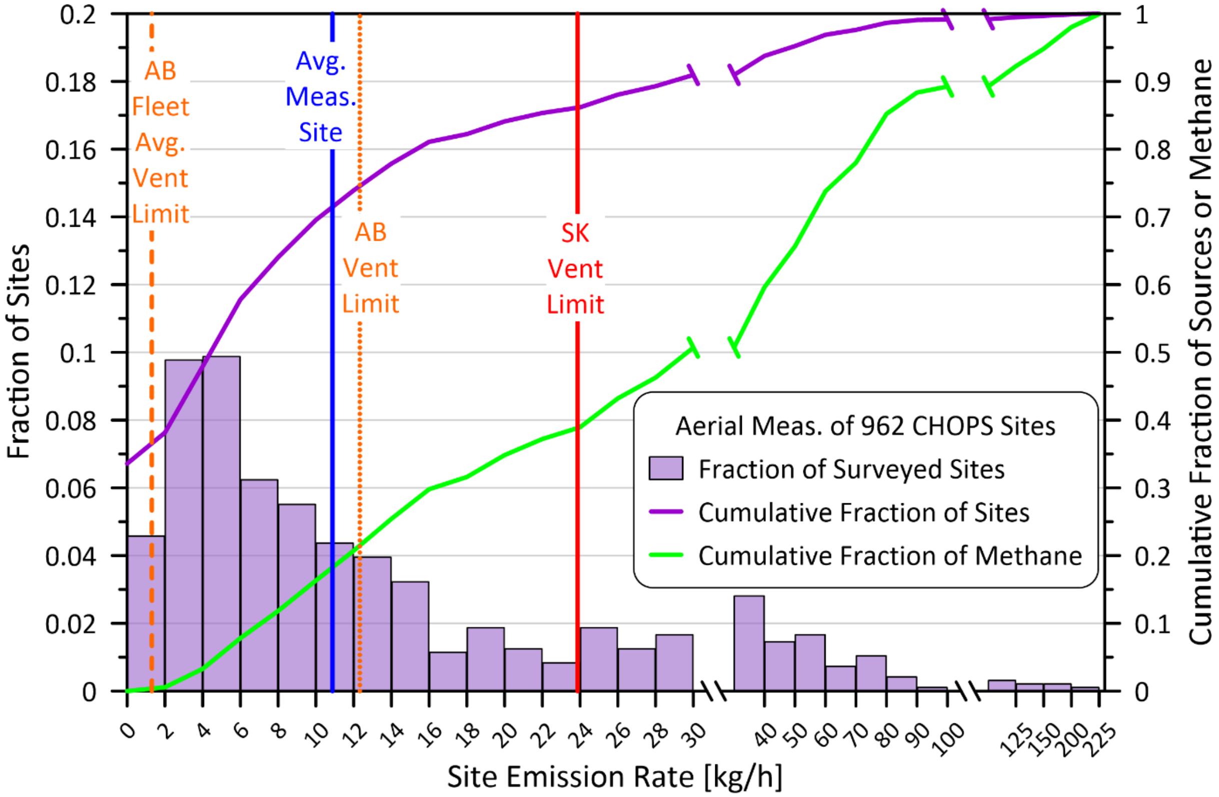

Figure S5: Histogram and cumulative distributions of measured methane emissions at the 962 sites covered by the aerial study (note the uneven scale on the horizontal axis to enable visualization of the otherwise highly-skewed distribution). The measured average emission rate of 10.9 kg/h for the 962 sites is indicated in blue as are the relevant Saskatchewan regulated venting limit and reference (stronger) limits from neighboring Alberta.

S6 Marginal Well Fractions and Potential Mitigation Considering Current and Future Prices on Carbon

Figure S6 shows the impact of different carbon prices (imposed as either a direct methane charge or a carbon pollution price) on the fraction of methane (Figure S6a) and oil (Figure S6b) coming from environmentally marginal wells (defined as where the cost of the methane emissions is equal to or greater than the value of the produced oil). Three different prices for the WCS oil blend were used: a low value of US$30/bbl, the October 2022 average value of US$66.38/bbl, and a high value of US$100/bbl. These values represent the approximate range of market price for the WCS blend over the past 5 years, ignoring any transitory corrections. As explained in the manuscript, this price exaggerates the net profit of each well as it ignores all operating costs, additional costs for CO_2 emissions, and the lower value of the oil as extracted at the wellhead compared to the WCS blend. Current and scheduled methane charges (in the US) and carbon pollution prices (in Canada) are included as vertical lines.

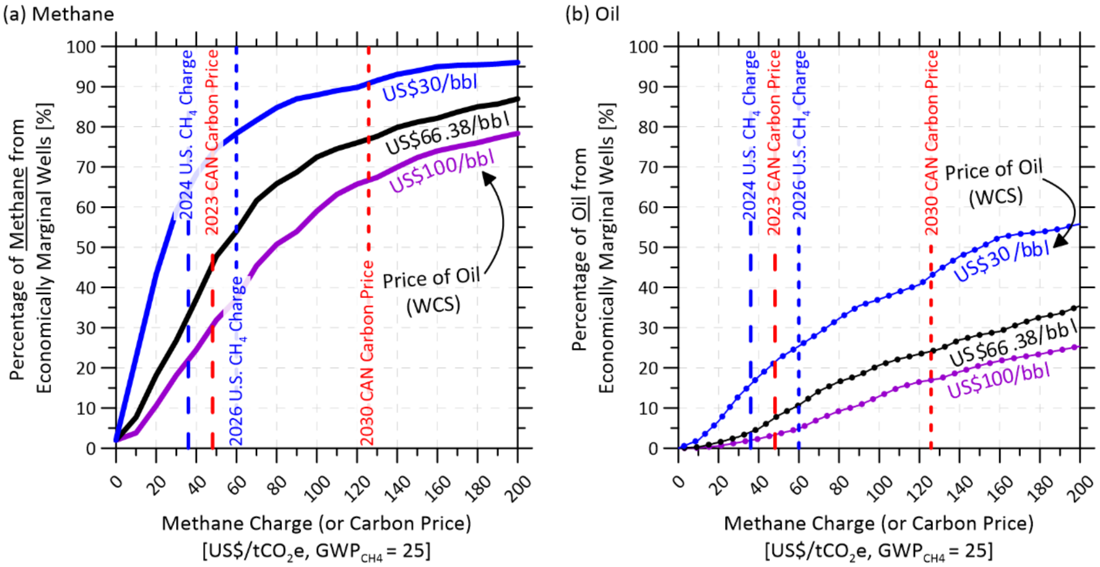

Figure S6: (a) Percentage of methane emissions from the 962 sites covered by the aerial study that are coming from economically marginal wells, as a function of a direct methane charge (or a price on carbon using a Global Warming Potential (GWP) for methane of 25). These results are presented for three different market prices of the WCS blend. (b) Corresponding curves for the percentage of oil coming from these same economically marginal wells. Current and future carbon pricing schemes are included as vertical lines for both the USA and Canada, using a current CAD/USD exchange rate of 0.74.

Although the results Figure S6 emphasize how many of these wells have low or even negative apparent value given their current methane emission rates, this is readily changed if the methane emissions are mitigated. Previous technoeconomic analysis of flaring and venting mitigation options in neighboring Alberta suggests that a range of commercially available mitigation solutions exist, including capturing gas for sale, combustion in auxiliary burners or heaters, or destruction in stand-alone combustors.[14] The latter is generally the simplest option to implement and discussions with one major supplier of combustors for upstream production sites in Western Canada suggests installed costs of CA$65,000 to 100,000 can be expected at the time of writing. Figure S7a shows that based on the avoided costs of methane emissions, 82% of emitting sites – representing 97% of the emitted methane in the sample – would have simple payback periods of less than 2 years assuming the maximum installed combustor cost of CA$100,000 and an applicable methane charge of CA$65/t$CO_2$e. Figure S7b shows that the mitigation costs are small relative to the value of the produced oil, with simple payback periods of less than 12 months for 98% of sites comprising 98% of methane from these producing sites (using an oil price of US$66.38/bbl). Notably, combustor solutions destroy any market value of the gas, whereas capture of the gas for sale into pipelines could offer better economics.

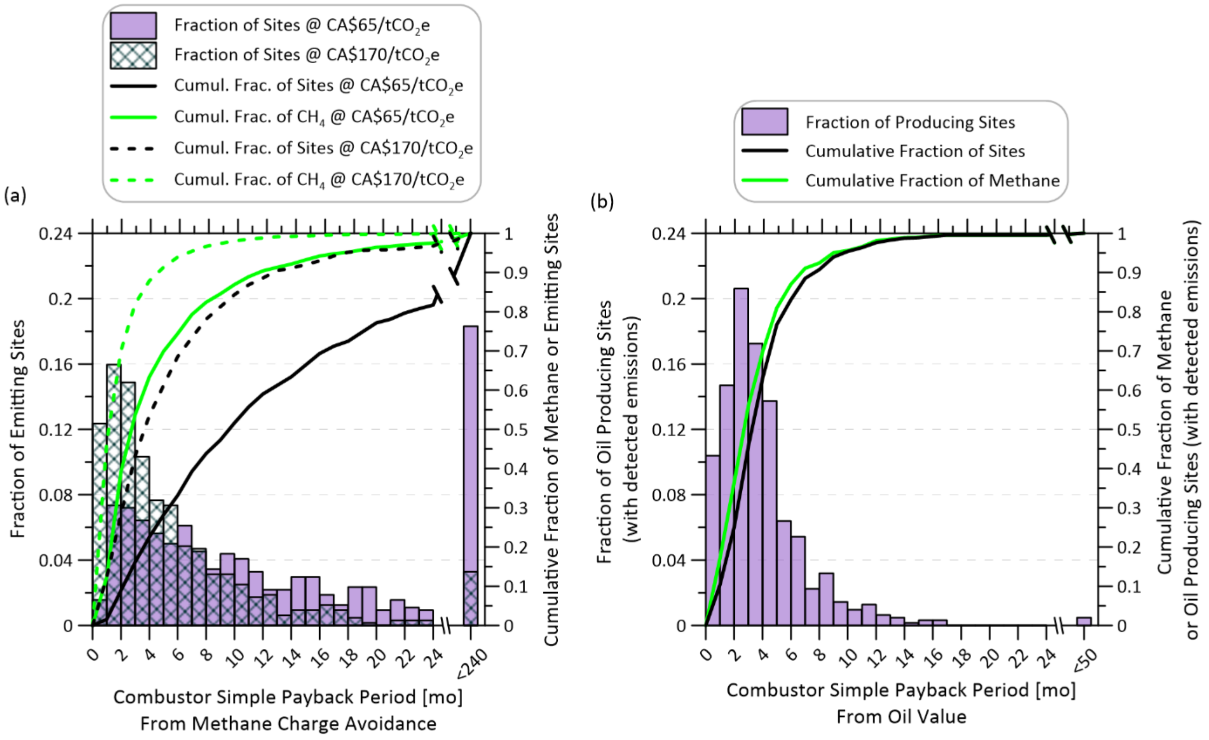

Figure S7: Simple payback periods for methane mitigation via an enclosed combustor (assumed installed cost of CA$100,000) at sites with detected emissions. (a) Payback periods and methane mitigation fraction based on avoiding assumed methane emission charge of either CA$65/t$CO_2$e or CA$170/tCO_2e. (b) Simple payback period relative to value of produced oil using the October 2022 market value of US$66.38/bbl.

S7 References

(1) Harper, K.; Lansing, J.; Dietz, T. Field Experience of Ultrasonic Flow Meter Use in CO2-Rich Applications. In *27th North Sea Flow Measurement Workshop*; Energy Institute: Tonsberg, Norway, 2009.

(2) Mills, C. *Flow Measurement in Support of Carbon Capture, Utilisation and Storage (CCUS)*; 2021_299; TUV SUD National Engineering Laboratory: Glasgow, UK, 2021.

(3) Warner, K.; Zanker, K. Noise Reduction Ultrasonic Gas Flow Measurement. In *4th International Symposium on Fluid Flow Measurement*; Denver, CO, 2015; pp 1–9.

(4) Dusseault, M. B. *CHOPS: Cold Heavy Oil Production with Sand in the Canadian Heavy Oil Industry*; Waterloo, 2002.

(5) Sentio Engineering. *Technology for Emissions Reductions: Cold Heavy Oil Production with Sand (CHOPS) Methods for Reduction of Methane Venting*; Petroleum Technology Alliance of Canada (PTAC), 2015.

(6) Campbell, J. M. *Gas Conditioning and Processing, Volume 1: The Basic Principles*, 7th ed.; Campbell Petroleum Series: Norman, OK, 1992.

(7) Hendler, A.; Nunn, J.; Lundeen, J. *VOC Emissions from Oil and Condensate Storage Tanks*; Austin, TX, 2009.

(8) U.S. EPA. AP-42: Chapter 7 Liquid Storage Tanks. Section 7.1 Organic Liquid Storage Tanks. **2020**, *1*, 203.

(9) Vaughn, T. L.; Bell, C. S.; Pickering, C. K.; Schwietzke, S.; Heath, G. A.; Pétron, G.; Zimmerle, D. J.; Schnell, R. C.; Nummedal, D. Temporal Variability Largely Explains Top-down/Bottom-up Difference in Methane Emission Estimates from a Natural Gas Production Region. *Proc. Natl. Acad. Sci.* **2018**, 201805687. https://doi.org/10.1073/pnas.1805687115.

(10) Schwietzke, S.; Petron, G.; Conley, S. A.; Pickering, C.; Mielke-Maday, I.; Dlugokencky, E. J.; Tans, P. P.; Vaughn, T. L.; Bell, C. S.; Zimmerle, D. J.; Wolter, S.; King, C.; White, A. B.; Coleman, T.; Bianco, L.; Schnell, R. Improved Mechanistic Understanding of Natural Gas Methane Emissions from Spatially-Resolved Aircraft Measurements. *Environ. Sci. Technol.* **2017**, acs.est.7b01810. https://doi.org/10.1021/acs.est.7b01810.

(11) Petrinex. Petrinex Public Data Access (Saskatchewan) https://www.petrinex.ca/PD/Pages/SPD.aspx (accessed Jan 9, 2023).

(12) SK MER. *Directive PNG036: Venting and Flaring Requirements*; Saskatchewan Ministry of Energy and Resources (SK MER): Regina, SK, 2020.

(13) AER. *Directive 060*; Alberta Energy Regulator (AER): Calgary, AB, 2021.

(14) Tyner, D. R.; Johnson, M. R. A Techno-Economic Analysis of Methane Mitigation Potential from Reported Venting at Oil Production Sites in Alberta. *Environ. Sci. Technol.* **2018**, *52* (21), 12877–12885. https://doi.org/10.1021/acs.est.8b01345.

S8 Underlying Data Tables

Table S3: Preview of supplemental aerial measurement data for all detected emissions, including a source description and associated site IDs. Full table provided in separate .xlsx file.

Anonymized Site ID	Anonymized Source ID	Source Description	MC-Average Emission Rate [kg/h]
SK_542	Src_1	Engine Shed	5.7
SK_933	Src_2	Tank	57.3
SK_960	Src_3	Tank	136.7
⋮	⋮	⋮	⋮
SK_609	Src_748	Wellhead (CHOPS)	2.5
SK_879	Src_749	Unknown equipment	31.6

Table S4: Preview of supplemental aerial measurement data for site-total emission rate and emission intensity. Full table provided in separate .xlsx file.

Anonymized Site ID	MC-Average Emission Rate (kg/h)	Methane Emission Intensity [gCO2e/MJ]
SK_1	0.0	0
SK_2	0.0	0
SK_3	0.0	0
⋮	⋮	⋮
SK_960	136.7	546
SK_961	179.6	317
SK_962	201.9	251

Table S5: Preview of supplemental ground measurement data provided for all eleven sites. Full time series for each site provided as individual sheets in separate .xlsx file.

Time - Excel, CST [days]	Methane Volume Fraction [%]	VentX Methane Mass Flow [kg/h]	VentX Flow Rate [m^3/day]	Ultrasonic Flow Rate [m^3/day]
44426.43175	89.5	44.0	1740.1	1424.6
44426.43177	89.4	41.8	1656.0	2026.6
44426.43179	89.4	42.5	1682.5	1872.8
44426.43181	89.7	44.0	1737.1	1359.3
44426.43183	89.4	43.6	1726.6	1303.7
⋮	⋮	⋮	⋮	⋮
44426.6975	92.1	53.5	2054.9	1509.3
44426.69759	92.0	53.1	2041.8	1908.4
44426.69762	92.0	52.9	2036.7	1929.7
